# A comparison of inflammation markers for predicting oncological outcomes after surgical resection of non-small-cell lung cancer: a validated analysis of 2,066 patients

**DOI:** 10.1038/s41598-020-76644-8

**Published:** 2020-11-11

**Authors:** Hsiang-Ling Wu, Yu-Ming Wu, Jui-Tai Chen, Kuang-Yi Chang, Yih-Giun Cherng, Shih-Pin Lin, Mei-Yung Tsou, Ying-Hsuan Tai

**Affiliations:** 1 Department of Anesthesiology, Taipei Veterans General Hospital, Taipei, Taiwan; 2 School of Medicine, National Yang-Ming University, Taipei, Taiwan; 3 Department of Anesthesiology, Shuang Ho Hospital, Taipei Medical University, No.291, Zhongzheng Rd., Zhonghe District, New Taipei City, 23561 Taiwan; 4 Department of Anesthesiology, School of Medicine, College of Medicine, Taipei Medical University, Taipei, Taiwan

**Keywords:** Biomarkers, Medical research, Oncology

## Abstract

Clinical and pathological predictors have proved to be insufficient in identifying high-risk patients who develop cancer recurrence after tumour resection. We aimed to compare the prognostic ability of various inflammation markers in patients undergoing surgical resection of lung cancer. We consecutively included 2,066 patients with stage I–III non-small-cell lung cancer undergoing surgical resection at the center between 2005 and 2015. We evaluated prognostic nutritional index, neutrophil-to-lymphocyte ratio, and platelet-to-lymphocyte ratio along with their perioperative changes. We conducted stepwise backward variable elimination and internal validation to compare the selected markers’ predictive performance for postoperative recurrence-free survival and overall survival. Preoperative neutrophil-to-lymphocyte ratio independently predicts recurrence-free survival (HR: 1.267, 95% CI 1.064–1.509, *p* = 0.0079, on base-2 logarithmic scale) and overall survival (HR: 1.357, 95% CI 1.070–1.721, *p* = 0.0117, on base-2 logarithmic scale). The cut-off value is 2.3 for predicting both recurrence (sensitivity: 46.1% and specificity: 66.7%) and mortality (sensitivity: 84.2% and specificity: 40.4%). Advanced cancer stage, poor tumour differentiation, and presence of perineural infiltration were significantly correlated with higher preoperative neutrophil-to-lymphocyte ratio. We concluded that preoperative neutrophil-to-lymphocyte ratio is superior to prognostic nutritional index and platelet-to-lymphocyte ratio in predicting postoperative recurrence and mortality of patients undergoing surgical resection of non-small-cell lung cancer.

## Introduction

An estimated 2.09 million newly diagnosed cases of lung cancer led to the highest death rates among all cancers worldwide in 2018^1^. Each year lung cancer accounts for 1.76 million deaths gloablly^1^. For operable tumours, surgical resection remains the potentially curative treatment for stage I through IIIA non-small-cell lung cancer (NSCLC)^2^. However, cancer relapse after tumour resection is common with the 3-year recurrence rate up to 20% reported in early-stage NSCLC^3^, which significantly impacts patients’ survival after surgery.

Systemic inflammation correlates closely with tumour invasion. Accumulating evidence has shown that the inflammation-based markers before or after cancer treatments can predict recurrence and survival in cancer patients^4,5^. For NSCLC, this has been reported for prognostic nutritional index (PNI)^5–9,15^, neutrophil-to-lymphocyte ratio (NLR)^4,10–20^, and platelet-to-lymphocyte ratio (PLR)^21^. Nevertheless, the prognostic performance of inflammation markers in NSCLC is not completely clarified because of conflicting results and study limitations, such as small sample size (< 1000 in most studies)^6,8–13,15,16,18–21^, inadequate adjustment for confounders^6,11,13^, no measurement of dynamic change of individual factors before and after surgery^6–13,15–21^, and no assessment of cancer recurrence^6,7,9–11^. Furthermore, most of previous studies did not compare different inflammation-based markers in their prognostic performance of NSCLC^6–11,14,16,18,19^, and therefore it remains unclear which marker serves as the superior prognostic factor for postoperative survival in NSCLC patients.

Accordingly, we performed the single-center cohort study to evaluate the prognostic ability of various inflammation-related markers with regard to recurrence and mortality after radical resection of NSCLC. Notably, we included a large cohort and a detailed list of covariates to examine various inflammation markers along with their dynamic changes before and after tumour resection to determine the optimal prognostic markers. We also performed the model validation to test the robustness of our findings.

## Results

In the 2,066 included patients, the median follow-up interval was 41.6 months (interquartile range 24.4–68.9). Table 1 showed the demographic, clinical and pathologic characteristics and distributions of the 10 inflammation markers of the derivation, validation, and entire cohort.

**Table 1:** Demographic, clinical and pathologic characteristics of the included patients.

	Derivation cohort (n = 1,008)	Validation cohort (n = 1,058)	Entire cohort (n = 2,066)
**Age, year**	64.3 ± 11.2	63.0 ± 11.2	63.6 ± 11.2
**Sex, male**	525 (52.1%)	530 (50.1%)	1055 (51.1%)
**Body mass index, kg·m**^**-2**^	24.0 ± 3.5	24.1 ± 3.5	24.0 ± 3.5
**Cigarette smoking**	284 (28.2%)	269 (25.4%)	553 (26.8%)
**ASA class ≥ 3**	264 (26.2%)	243 (23.0%)	507 (24.5%)
**ECOG grade ≥ 1**	345 (34.2%)	298 (28.2%)	643 (31.1%)
**Comorbidities**			
Chronic obstructive pulmonary disease	111 (11.0%)	97 (9.2%)	208 (10.1%)
Diabetes	173 (17.2%)	161 (15.2%)	334 (16.2%)
Coronary artery disease	105 (10.4%)	97 (9.2%)	202 (9.8%)
Heart failure	51 (5.1%)	35 (3.3%)	86 (4.2%)
Stroke	38 (3.8%)	35 (3.3%)	73 (3.5%)
Chronic kidney disease	83 (8.2%)	84 (7.9%)	167 (8.1%)
**Preoperative pulmonary function**			
FVC, % predicted	87.1 ± 15.8	87.5 ± 15.4	87.3 ± 15.6
FEV1, % predicted	85.7 ± 16.4	86.1 ± 16.4	85.9 ± 16.4
**Preoperative carcinoembryonic antigen, μg·L**^-1^*	2.4 (1.8–3.9)	2.3 (1.7–3.7)	2.4 (1.8–3.8)
**Preoperative hemoglobin concentration, g·dL**^**-1**^	13.0 ± 1.5	13.1 ± 1.4	13.1 ± 1.5
**Surgical and anesthetic variables**			
*Type of surgery*			
Sublobar resection or lobectomy	769 (76.5%)	799 (75.7%)	1568 (76.1%)
Bilobectomy or pneumonectomy	236 (23.5%)	257 (24.3%)	493 (23.9%)
*Thoracoscopic surgery*	642 (63.7%)	786 (74.3%)	1428 (69.1%)
*Radical lymph node dissection*	902 (89.7%)	797 (75.4%)	1699 (82.4%)
*Intraoperative blood loss, mL*	100 (50–200)	50 (30–150)	100 (30 200)
*Blood transfusion*	168 (16.7%)	91 (8.6%)	259 (12.5%)
*Epidural analgesia*	838 (83.1%)	864 (81.7%)	1702 (82.4%)
*Anesthesia time, min*	315 (270–375)	300 (240–360)	300 (255–360)
**Inflammation biomarkers**			
Prognostic nutritional index	50.2 (46.9–53.4)	49.5 (45.6–52.9)	49.8 (46.3–53.1)
Preoperative NLR	2.0 (1.4–2.7)	1.9 (1.4–2.6)	1.9 (1.4–2.7)
Postoperative NLR	12.4 (8.7–17.9)	11.9 (8.8–17.9)	12.2 (8.7–17.9)
Absolute change of NLR	10.2 (6.5–15.4)	9.8 (6.6–15.3)	10.0 (6.6–15.3)
Relative change of NLR, %	525 (308–822)	517 (302–814)	521 (305–817)
Preoperative PLR	123.7 (94.6–164.9)	121.5 (95.7–156.1)	122.6 (95.0–162.1)
Postoperative PLR	227.7 (162.3–323.5)	223.6 (160.9–314.6)	225.2 (161.9–320.2)
Absolute change of PLR	97.4 (40.6–178.0)	92.3 (42.9–170.0)	96.1 (41.9–177.3)
Relative change of PLR, %	79 (36–148)	82 (33–145)	81 (36–146)
Absolute change of lymphocyte count, 10^3^·μL^−1^	− 863 (− 1225 to − 515)	− 1223 (− 1751 to − 729)	− 1019 (− 1496 to − 608)
**Pathologic features**			
*Cancer stage*			
I	708 (70.2%)	789 (74.6%)	1497 (72.5%)
II	135 (13.4%)	114 (10.8%)	249 (12.1%)
III	165 (16.4%)	155 (14.7%)	320 (15.5%)
*Subtype*			
Adenocarcinoma	821 (81.5%)	895 (84.6%)	1716 (83.1%)
Squamous cell carcinoma	130 (12.9%)	113 (10.7%)	243 (11.8%)
Others	57 (5.7%)	50 (4.7%)	107 (5.2%)
*Tumor differentiation*			
Good	73 (7.3%)	132 (12.5%)	205 (9.9%)
Moderate	610 (60.6%)	630 (59.6%)	1240 (60.1%)
Poor	324 (32.2%)	295 (27.9%)	619 (30.0%)
*Microscopic necrosis*	248 (24.6%)	202 (19.1%)	450 (21.8%)
*Lymphocytic infiltration*	113 (11.2%)	96 (9.1%)	209 (10.1%)
*Lymphovascular invasion*	306 (30.4%)	292 (27.6%)	598 (28.9%)
*Perineural infiltration*	36 (3.6%)	32 (3.0%)	68 (3.3%)
**Preoperative chemotherapy ± radiotherapy**	51 (5.1%)	45 (4.3%)	96 (4.7%)
**Postoperative chemotherapy**	483 (47.9%)	463 (43.8%)	946 (45.8%)
**Postoperative radiotherapy**	63 (6.3%)	51 (4.8%)	114 (5.5%)
**Year of operation**			
2005–2010	500 (49.6%)	365 (34.5%)	865 (41.9%)
2011–2015	508 (50.4%)	693 (65.5%)	1201 (58.1%)

Values are mean ± SD, count (percent), or median (interquartile range).

ASA, American Society of Anesthesiologists; ECOG, Eastern Cooperative Oncology Group; FEV1, forced expiratory volume in one second; FVC, forced vital capacity; NLR, neutrophil-to-lymphocyte ratio; PLR, platelet-to-lymphocyte ratio.

### Prognostic factors for cancer recurrence

Supplementary Table 1 showed the results of univariate analysis for recurrence-free survival. The multivariant analysis of derivation cohort showed preoperative NLR was the only independent predictor for recurrence-free survival among the 10 included inflammation markers. Higher preoperative PNI predicted a higher risk of cancer recurrence, hazard ratio (HR): 1.267 (95% CI 1.064–1.509, on base-2 logarithmic scale). Other independent prognostic factors for recurrence were age (HR: 1.012), diabetes (HR: 1.361), cancer stage (II vs. I, HR: 1.612; III vs. I, HR: 2.418), tumour differentiation (moderate vs. good, HR: 5.669; poor vs. good, HR: 9.647), lymphovascular invasion (HR: 2.118), postoperative chemotherapy (HR: 1.582), and postoperative radiotherapy (HR: 1.437) (Table 2).

**Table 2:** Backward variable selection for recurrence-free survival and overall survival (derivation cohort, n = 1,008).

Recurrence-free survival	HR (95% CI)	*p*	Overall survival	HR (95% CI)	*p*
**Preoperative NLR**‡	1.267 (1.064–1.509)	0.0079	**Preoperative NLR**‡	1.357 (1.070–1.721)	0.0117
**Age**	1.012 (1.002–1.023)	0.0231	**Age**	1.031 (1.012–1.050)	0.0011
**Diabetes**	1.361 (1.040–1.783)	0.0249	**ECOG grade ≥ 1**	1.495 (1.011–2.211)	0.0439
**Cancer stage**		< .0001	**Preoperative CEA level†**	1.830 (1.206–2.778)	0.0045
II versus I	1.612 (1.175–2.210)	0.0031	**Cancer stage**		< .0001
III versus I	2.418 (1.782–3.280)	< .0001	II versus I	1.985 (1.233–3.195)	0.0048
**Tumor differentiation**		< .0001	III versus I	3.086 (1.979–4.812)	< .0001
Moderate versus good	5.669 (1.394–23.049)	0.0153	**Lymphovascular invasion**	2.006 (1.361–2.956)	0.0004
Poor versus good	9.647 (2.357–39.473)	0.0016	**Microscopic necrosis**	1.473 (1.032–2.103)	0.0330
**Lymphovascular invasion**	2.118 (1.653–2.713)	< .0001	**Anesthesia time**‡	1.702 (1.020–2.841)	0.0418
**Postoperative chemotherapy**	1.582 (1.195–2.094)	0.0014			
**Postoperative radiotherapy**	1.437 (1.023–2.019)	0.0365			

HR, hazard ratio; CI, confidence interval; CEA, carcinoembryonic antigen; ECOG, Eastern Cooperative Oncology Group; NLR, neutrophil-to-lymphocyte ratio.

^‡^On base-2 logarithmic scale.

^†^On base-10 logarithmic scale.

**Figure 1:**
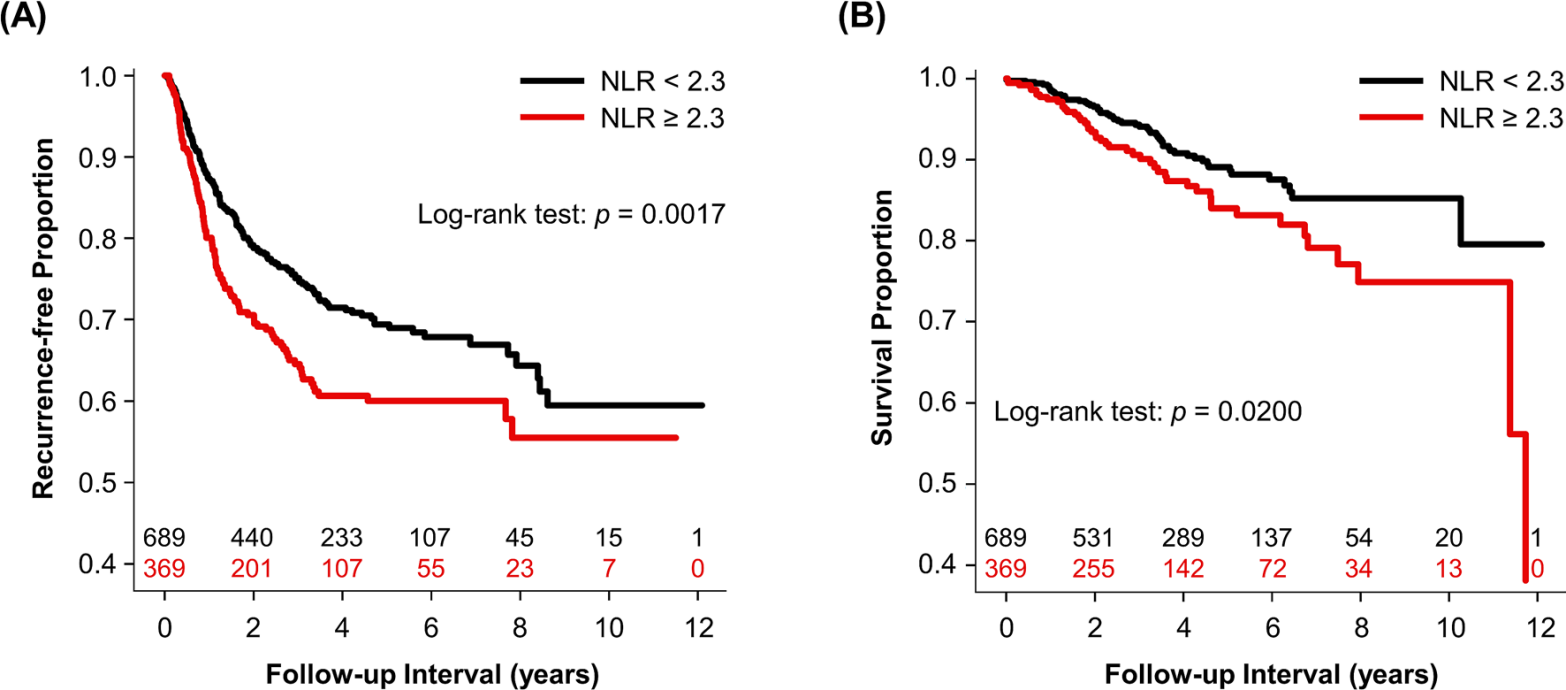
Kaplan–Meier curves for (**A**) recurrence-free survival and (**B**) overall survival for dichotomous preoperative neutrophil-to-lymphocyte ratio with number of subjects at risk (validation cohort, n = 1,058).

Youden's index of receiver operating characteristic (ROC) curves determined the cut-off values of preoperative NLR as 2.3 for predicting 1-year recurrence (sensitivity: 46.1%; specificity: 66.7%). The 1-year, 3-year, and 5-year recurrence-free survival rates were 87.3% (95% CI 84.8–89.8), 75.1% (95% CI 71.6–78.6), 69.4% (95% CI 65.3–73.5) for patients with preoperative NLR < 2.3 and 80.0% (95% CI 75.9–84.1), 64.6% (95% CI 59.3–69.9), 60.0% (95% CI 54.3–65.7) for those with preoperative NLR ≥ 2.3. Figure 1A is the Kaplan–Meier curves for recurrence-free survival for dichotomous NLR. Table 3 and supplementary Table 3 show the c-statistics of preoperative NLR as continuous and dichotomous variables for predicting cancer recurrence. Of note, the results of patients with epidermal growth factor receptor (EGFR) mutation-positive NSCLC were similar to the overall results.

**Table 3:** C-statistics of preoperative neutrophil-to-lymphocyte ratio for predicting recurrence and mortality (validation cohort, n = 1,058).

	Continuous NLR		Dichotomous NLR†	
	C-statistic (95% CI)	*p*	C-statistic (95% CI)	*p*
1-Year recurrence	0.550 (0.498–0.602)	0.0484	0.562 (0.512–0.612)	0.0140
3-Year recurrence	0.563 (0.522–0.603)	0.0021	0.554 (0.514–0.594)	0.0077
5-Year recurrence	0.558 (0.519–0.597)	0.0033	0.548 (0.509–0.587)	0.0143
1-Year mortality	0.610 (0.485–0.734)	0.1012	0.564 (0.431–0.696)	0.3415
3-Year mortality	0.601 (0.527–0.676)	0.0078	0.558 (0.483–0.634)	0.1246
5-Year mortality	0.563 (0.500–0.626)	0.0477	0.546 (0.483–0.609)	0.1465

CI, confidence interval; NLR, neutrophil-to-lymphocyte ratio.

^†^The cut-off value is 2.3 for both recurrence and mortality.

### Prognostic factors for all-cause mortality

Supplementary Table 2 showed the results of univariate analysis for overall survival. Backward variable elimination procedures demonstrated preoperative NLR was the only independent predictor among the included inflammation markers for overall survival (HR: 1.357, 95% CI 1.070–1.721, on base-2 logarithmic scale). Other significant prognostic factors for all-cause mortality were age (HR: 1.031), ECOG grade ≥ 1 (HR: 1.495), preoperative carcinoembryonic antigen (CEA) level (HR: 1.830, on base-10 logarithmic scale), cancer stage (II vs. I, HR: 1.985; III vs. I, HR: 3.086), microscopic necrosis (HR: 1.473), lymphovascular invasion (HR: 2.006), and anesthesia time (HR: 1.702, on base-2 logarithmic scale). Based on the Youden's index of the ROC curve, we determined the cut-off point of preoperative NLR as 2.3 for predicting 1-year mortality (sensitivity: 84.2%; specificity: 40.4%). The 1-year, 3-year, and 5-year survival rates were 98.5% (95% CI 97.5–99.5), 94.3% (95% CI 92.3–96.3), 89.1% (95% CI 86.2–92.0) for patients with preoperative NLR < 2.3 and 97.4% (95% CI 95.8–99.0), 90.6% (95% CI 87.3–93.9), 84.0% (95% CI 79.1–88.9) for those with preoperative NLR ≥ 2.3. Figure 1B shows the Kaplan–Meier curves of overall survival for dichotomous NLR. Table 3 and supplementary Table 3 show the c-statistics of continuous and dichotomous preoperative NLR for predicting all-cause mortality.

### Factors associated with preoperative NLR

We found 9 factors significantly associated with preoperative NLR, including sex, American Society of Anesthesiologists (ASA) class ≥ 3, percentage of predicted forced vital capacity (FVC) and forced expiratory volume in one-second (FEV1), chronic kidney disease, preoperative haemoglobin concentration, cancer stage, tumour differentiation, and perineural infiltration. (Table 4) In univariate analysis, cigarette smoking was associated with a higher level of preoperative NLR (beta: 0.508, 95% CI 0.347–0.669, *p* < 0.0001), but the association was no longer significant after the adjustment for other covariates.

**Table 4:** Baseline factors associated with preoperative neutrophil-to-lymphocyte ratio (entire cohort, n = 2,066).

	Beta (95% CI)	*p*
Sex, male	0.397 (0.225–0.568)	< .0001
ASA class ≥ 3	0.258 (0.087–0.428)	0.0031
Preoperative FVC, % predicted	0.907 (0.077–1.738)	0.0323
Preoperative FEV1, % predicted	− 1.496 (− 2.265 to − 0.726)	0.0001
Chronic kidney disease	0.510 (0.242–0.778)	0.0002
Preoperative hemoglobin concentration	− 0.065 (− 0.120 to − 0.010)	0.0205
Advanced cancer stage	0.126 (0.025–0.226)	0.0140
Poor tumor differentiation	0.274 (0.126–0.422)	0.0003
Perineural infiltration	0.459 (0.054–0.864)	0.0263

CI, confidence interval; ASA, American Society of Anesthesiologists; FEV1, forced expiratory volume in one second; FVC, forced vital capacity.

### Correlation between inflammation markers

Table 5 shows the correlation coefficients between different types of inflammation markers. Of note, preoperative NLR was inversely correlated with PNI and positively correlated with other inflammation markers.

**Table 5:** Correlation between different types of inflammation markers (derivation cohort, n = 1,008).

	NLR-1	PLR-1	NLR-2	PLR-2	PNI	Change of lymphocyte
NLR-1	1.000	0.672 (*p* < 0.0001)	0.094 (*p* = 0.0003)	0.093 (*p* = 0.0003)	− 0.395 (*p* < 0.0001)	0.286 (*p* < 0.0001)
PLR-1	0.672 (*p* < 0.0001)	1.000	0.094 (*p* = 0.0002)	0.149 (*p* < 0.0001)	− 0.402 (*p* < 0.0001)	0.353 (*p* < 0.0001)
NLR-2	0.094 (*p* = 0.0003)	0.094 (*p* = 0.0002)	1.000	0.789 (*p* < 0.0001)	− 0.065 (*p* = 0.0113)	− 0.119 (*p* < 0.0001)
PLR-2	0.093 (*p* = 0.0003)	0.149 (*p* < 0.0001)	0.789 (*p* < 0.0001)	1.000	− 0.096 (*p* = 0.0002)	− 0.131 (*p* < 0.0001)
PNI	− 0.395 (*p* < 0.0001)	− 0.402 (*p* < 0.0001)	− 0.065 (*p* = 0.0113)	− 0.096 (*p* = 0.0002)	1.000	− 0.322 (*p* < 0.0001)
Change of lymphocyte	0.286 (*p* < 0.0001)	0.353 (*p* < 0.0001)	− 0.119 (*p* < 0.0001)	− 0.131 (*p* < 0.0001)	− 0.322 (*p* < 0.0001)	1.000

Values are Pearson correlation coefficients.

PNI, prognostic nutritional index; NLR-1, preoperative neutrophil-to-lymphocyte ratio; NLR-2, postoperative neutrophil-to-lymphocyte ratio; PLR-1, preoperative platelet-to-lymphocyte ratio; PLR-2, postoperative platelet-to-lymphocyte ratio.

## Discussion

Our results showed preoperative NLR independently predicts both postoperative cancer recurrence and all-cause mortality after surgical resection of NSCLC. Additionally, we also discovered NLR is superior to PLR and PNI in the prognostic performance for oncological outcome. Higher preoperative NLR reflects tumours with advanced stage, worse differentiation and greater invasion. Compared with previous studies, our study has several strengths to assess the predictive value of inflammation markers. First, we used a large patient sample to allow for sufficient statistical power. Second, we included a comprehensive collection of clinical and pathological variables to minimize potential confounding effects. Third, we conducted an internal validation to assess the predictive performance of the selected predictor. Our results provided valuable evidence for preoperative risk stratification and postoperative individualized anti-cancer therapy and surveillance in patients with NSCLC.

Prior studies have reported a variety of inflammation markers as predictors for oncological outcomes in multiple types of cancer^4–24^. In NSCLC, preoperative NLR, PLR and PNI have been demonstrated to predict cancer recurrence or mortality after tumour resection^4–21^. However, there is little evidence regarding the superior prognostic factor among these inflammation markers in the existing literature. In addition to preoperative NLR, PLR and PNI, our analysis included postoperative markers and dynamic changes in the stepwise model selection procedure. Our analyses indicated that preoperative NLR significantly associated with postoperative recurrence and mortality instead of PLR or PNI. Besides, preoperative NLR has better performance in predicting all-cause mortality rather than cancer recurrence. Difference in the included inflammation markers and covariates possibly explains the inconsistent findings between our study and others^4–21^. Prior study has showed NLR was inversely correlated with PNI and both factors were highly associated with patients’ clinical and pathological characteristics^15^, in agreement with our results. Our analyses have adjusted for a detailed list of covariates, and preoperative NLR remained significantly associated with cancer recurrence and all-cause mortality. Absolute change of lymphocyte count and dynamic change of NLR have also been reported to predict outcomes in liver cancer and colon cancer^22,24^. However, we did not confirm the prognostic values of these indexes in NSCLC. We used the derivation cohort and conducted the stepwise backward variable elimination to identify significant predictors while achieving goodness of model fit. Backward elimination has the advantage to evaluate the joint predictive performance of variables^25^. However, it has no capacity to identify less predictive individual variables that may not enter the model to demonstrate their combined effect^25^. Additionally, we performed an internal validation to test the robustness of our findings. The development and validation processes are important for prediction modelling^26^, which was lack in most previous studies^6–21^.

Several possible mechanisms may be helpful for explaining the relationship between preoperative NLR and postoperative NSCLC outcomes. First, systemic inflammation may activate the recruitment of regulatory T lymphocytes, enhance the levels of tumour necrosis factor alpha and interleukin-6, and trigger neutrophilia^27^. These responses may facilitate the growth and spread of cancer cells^27^. Seconds, in human non-small-cell lung cancer, tumour cells release granulocyte colony-stimulating factor and increase neutrophils in the peripheral blood^28^. Plasma level of granulocyte colony-stimulating factor was reported to predict shorter survival in NSCLC patients^29^. Third, higher NLR is associated with worse nutrition status^30^, which may affect immune system negatively, impair functional capacity, and cause inferior oncological outcomes after tumour resection.

Our analysis demonstrated that the NLR before surgical resection of NSCLC might reflect the invasiveness of tumours. Higher NLR was associated with advanced cancer stage, which is consistent with the results of a current meta-analysis^4^. Our results further showed preoperative NLR was also linked to worse differentiation, and presence of perineural infiltration. Patients with higher NLR might have tumours overexpressing granulocyte-colony stimulating factor, which also reflects the metastatic potential of cancer cells^31^. In addition, our study also demonstrated several clinical factors correlated with preoperative NLR, including chronic kidney disease and preoperative level of haemoglobin. Patients with chronic kidney disease are predisposed to compromised nutritional status^32^. Similarly, haemoglobin concentration is closely related to nutrition status and inflammatory response^33^. These findings offer a new insight into the role of inflammatory response and immune-nutritional status in the development of cancer.

In this study, univariate analysis showed cigarette smoking was associated with a higher preoperative NLR. However, the association disappeared after controlling for other covariates. It is well-established that cigarette smoke triggers inflammatory responses and suppresses the function of immune system in humans^34^. Cigarette smoking suppresses certain T-helper type 1 responses, while enhancing T-helper type 2 inflammation^34^. Our multivariable analysis showed that cigarette smoking did not correlate with higher recurrence or worse survival, contrasting with some previous studies^35^. Because our analyses included a variety of critical clinical and pathological variables for cancer outcome, the effect of cigarette smoking on postoperative prognosis may be overridden.

This study has the strengths of a large sample size, detailed collection of covariates, comprehensive analyses of various inflammation markers, and model validation. There are some limitations in this study. First, the study is retrospective, and we cannot further control for unrecorded variables. Second, our analysis did not include C-reactive protein (CRP), which was not a routine test for patients with NSCLC at the center. Therefore, we cannot compare the predictive values of CRP-based prognostic factors (e.g. Glasgow Prognostic Score and Prognostic Index) with other inflammation markers^36^. Third, this study is single-center, and therefore our results may not be generalizable to hospitals with different clinical settings. Fourth, we did not have detailed data about the regimen and cycle of chemotherapy for the included patients. Finally, residual confounding bias is always possible, although we have adjusted for many potential confounders.

In conclusion, preoperative NLR is superior to PNI and PLR in predicting postoperative recurrence and mortality in patients undergoing surgical resection of non-small-cell lung cancer. Additionally, higher preoperative neutrophil-to-lymphocyte ratio reflected advanced cancer stage, poor tumour differentiation, and presence of perineural infiltration. These results provided scientific evidence for identifying patients with high risk of cancer relapse and establishing individualized anti-cancer strategy after surgical resection. Our findings await more validations of future studies.

## Methods

This study was approved by the Institutional Review Board, Taipei Veterans General Hospital, Taiwan (IRB-TPEVGH No. 2015-11-010CC and No. 2018-06-009CC). The written informed consent was waived by the Institutional Review Board (chair: Professor Fa-Yauh Lee), and the whole datasets were anonymized and de-identifed before analysis. All methods of this study were performed in accordance with the relevant guidelines and regulations.

### Criteria of patient inclusion

**Figure 2:**
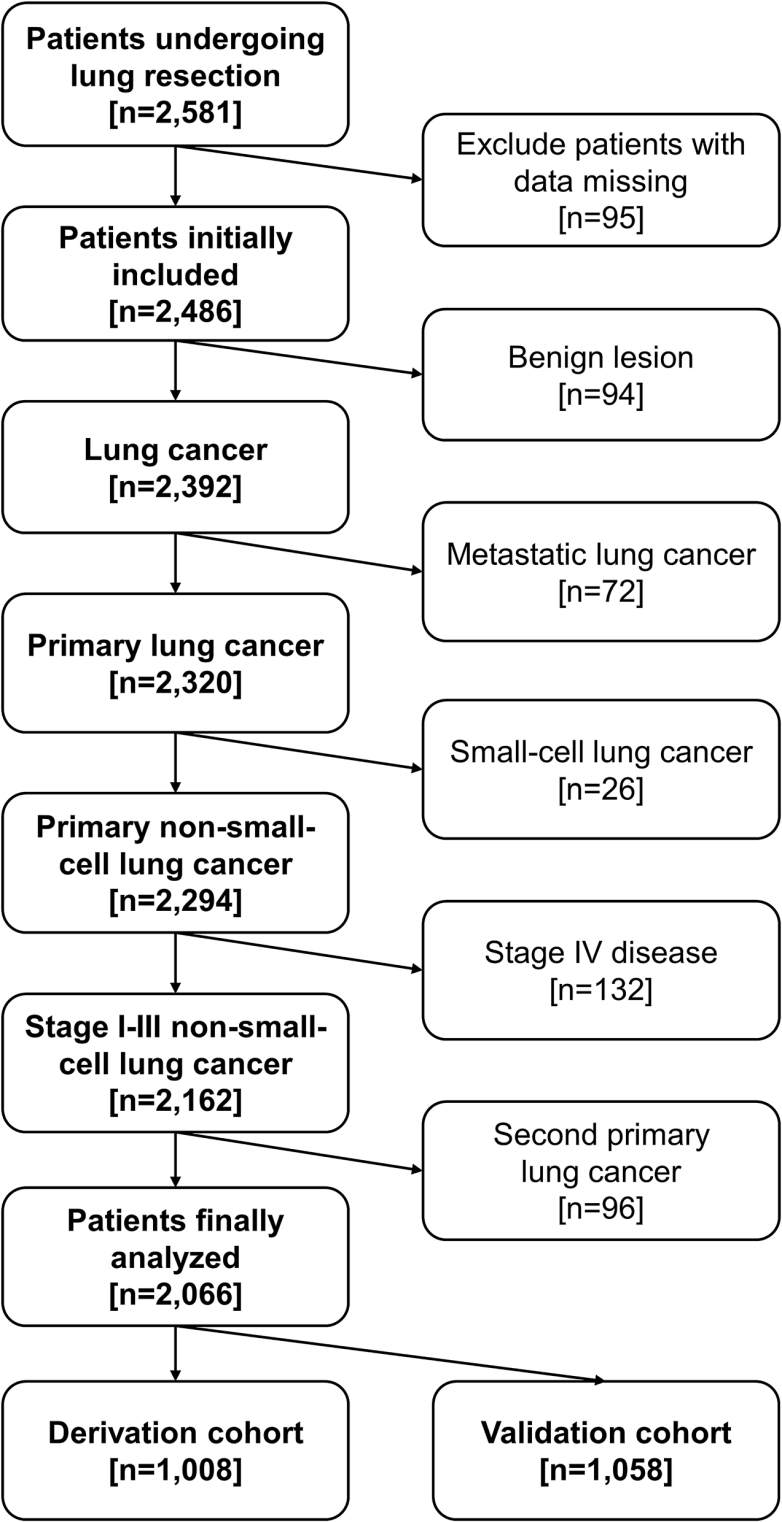
Flow diagram for patient selection.

We used the electronic medical databank of the medical center and consecutively collected 2,581 patients undergoing lung resection from 2005 to 2015. Patients were excluded if they had data missing, benign lung lesions, metastatic lung cancer, small-cell lung cancer, or distant metastatic disease diagnosed at the time of surgery. We also excluded patients who developed histology-confirmed second primary lung cancer after surgery during the follow-up interval. (Fig. 2) A total of 2,066 patients with stage I through III NSCLC were selected for further analyses.

### Inflammation markers for comparisons

We retrospectively collected the serum levels of albumin, neutrophil, lymphocyte, and platelet in peripheral blood one day before operation. Neutrophil, lymphocyte and platelet concentrations were also measured one day after operation. We included a total of 10 inflammation-based markers in the comparative analyses, as follows: prognostic nutritional index was calculated from 10 × serum albumin (g·dL^−1^) + 0.005 × lymphocyte count (10^3^ μ L^−1^)^5^. Neutrophil-to-lymphocyte ratio equals to neutrophil count/lymphocyte count. Similarly, platelet-to-lymphocyte ratio equals to platelet count/lymphocyte count. In addition to PNI, preoperative and postoperative NLR and PLR, we also analysed perioperative absolute change (postoperative value–preoperative value) and relative change [(postoperative value − preoperative value)/preoperative value] of NLR and PLR, and absolute change of lymphocyte count to compare their prognostic ability for NSCLC outcomes.

### Surgical resection and postoperative surveillance

At this center, all lung resections were done by experienced thoracic surgeons who performed at least 50 cases a year. Surgical resection of the included patients was intended for cure of NSCLC. After radical resection, patients were selected and received adjunct therapy with a previously described standard protocol^37^. We used cisplatin and carboplatin-based chemotherapy with or without radiation therapy based on cancer stage and pathology features^37^. We defined the adjuvant chemotherapy or radiation therapy as any treatments given within 90 days before or after surgery.

The routine surveillance after surgical resection included chest computed tomography every 6 months during the first two years after surgery and annually thereafter. Patients underwent magnetic resonance imaging, bone scintigraphy or positron emission tomography when locoregional recurrence or distant metastasis was suspected. Recurrent diseases were treated with a second resection, chemotherapy, radiation therapy either alone or in combination based on the pattern of recurrence, residual pulmonary functional reserve, and patients’ general condition.

### Collection of covariates

In prediction modelling for cancer recurrence and mortality, we selected the variables based on data availability, physiological plausibility and the existing literature. We used the electronic medical databank to collect potential confounding factors for NSCLC outcome^38^. Clinical variables included cigarette smoking, Eastern Cooperative Oncology Group (ECOG) grade, co-existing diseases, preoperative FVC and FEV1^39^, preoperative levels of CEA. Surgical and anaesthetic variables were extent of resection (sublobar resection, lobectomy, bilobectomy, or pneumonectomy), uses of thoracoscopic surgery, radical lymph node dissection, intraoperative blood loss, perioperative blood transfusion (during or within 7 days after surgery)^40,41^, and epidural analgesia^38,42^. Pathologic variables were cancer stage, subtype, tumour differentiation, microscopic necrosis, lymphocytic infiltration, lymphovascular invasion, and perineural infiltration^43,44^. We also recorded lung cancer with EGFR mutation.

### Measurement of recurrence and death

Primary outcome was recurrence-free survival, defined as the interval between the date of surgery and the date of first recurrence. Recurrence was determined by the presence of localized or metastatic deposits detected by imaging studies. Secondary outcome was overall survival, defined as the interval between the date of surgery to the date of death. The date of death was determined based on medical record and death certificate. For those without any events of recurrence or death, survival times were considered as the corresponding censored observations with the last visit date used as the censored date. Patient’s status was followed up until May 31, 2017.

### Statistical analysis

According to Schoenfeld’s formula for sample size estimation of proportional hazards models, at least 175 events are needed to attain a power of 0.8 assuming an alpha level of 0.05, HR of recurrence 1.55 and proportion of high-NLR group 37.1% (374/1008 in derivation cohort)^4,45^. In the derivation cohort, a total of 377 recurrences (212 and 165 in low-NLR and high-NLR groups, respectively) occurred during the study period, which has met the requirement of sample size.

Shapiro–Wilk test and Kolmogorov–Smirnov test were used as normality tests. Normally distributed variables were presented as mean with standard deviation. Non-normally distributed data were presented as median with interquartile range and logarithmic transformation was conducted to reduce skewness in the following analyses. We randomly split patients into derivation cohort (n = 1,008) and validation cohort (n = 1,058). We used the derivation cohort and performed univariate Cox proportional hazards regression to analyse the association of the inflammation markers and covariates with recurrence-free survival and overall survival. Significant variables in the univariate models were incorporated into the stepwise backward variable elimination process based on minimisation of the Akaike's Information Criterion (AIC) with a *p* value threshold of 0.05. We then used the validation dataset to assess the predictive ability of the selected inflammation marker with no additional variable selection or model fitting. Predictive performance was assessed by the c-statistic in the validation dataset. The optimal cut-off values of inflammation markers were determined as the threshold value with the joint maximum sensitivity and specificity of the ROC curves associated with the outcome of interest (Youden's index)^46^. Furthermore, we also performed stratified analyses for patients with EGFR mutation-positive NSCLC. Finally, backward model selection analyses were applied to determine the baseline clinical and pathological factors significantly correlated with the selected inflammation marker. We considered *p* < 0.05 statistically significant. All the statistical analyses were performed using IBM SPSS Statistics, Version 23.0 (IBM Corp., Armonk, NY, USA).

## Supplementary information

Supplementary Information.
