# Supplementary material for: A comparison of inflammation markers for predicting oncological outcomes after surgical resection of non-small-cell lung cancer: a validated analysis of 2,066 patients
Source: Sci Rep. 2020 Nov 11;10:19523. doi: 10.1038/s41598-020-76644-8 (PMC7658207; doi:10.1038/s41598-020-76644-8)
Supplement: Supplementary file 1 — Supplementary Information. [file 41598_2020_76644_MOESM1_ESM.doc]

**A comparison of inflammation markers for predicting oncological outcomes after surgical resection of non-small-cell lung cancer: A validated analysis of 2,066 patients**

Hsiang-Ling Wu, M.D.^1,2^, Yu-Ming Wu, M.D.^3,4^, Jui-Tai Chen, M.D., Ph.D.^3,4^, Kuang-Yi Chang, M.D., Ph.D.^1,2^, Yih-Giun Cherng, M.D., M.Sc.^3,4^, Shih-Pin Lin, M.D., Ph.D.^1,2^, Mei-Yung Tsou, M.D., Ph.D.^1,2^, Ying-Hsuan Tai, M.D., M.Sc.^1,2,3,4,†^

^1^ Department of Anesthesiology, Taipei Veterans General Hospital, Taipei, Taiwan; ^2^ School of Medicine, National Yang-Ming University, Taipei, Taiwan; ^3^ Department of Anesthesiology, Shuang Ho Hospital, Taipei Medical University, New Taipei City, Taiwan; ^4^ Department of Anesthesiology, School of Medicine, College of Medicine, Taipei Medical University, Taipei, Taiwan

† Corresponding author: Dr. Ying-Hsuan Tai; e-mail: [tp16960@gmail.com](mailto:tp16960@gmail.com)

Department of Anaesthesiology, Shuang Ho Hospital, Taipei Medical University, No.291, Zhongzheng Rd., Zhonghe District, New Taipei City, 23561, Taiwan. Tel: +886-2-27361661 ext. 3229; Fax: +886-2-27390500

**E-mails of co-authors**:

Hsiang-Ling Wu: [xianling30@gmail.com](mailto:xianling30@gmail.com); Yu-Ming Wu: b101096087@yahoo.com.tw;

Jui-Tai Chen: [19240@s.tmu.edu.tw](mailto:19240@s.tmu.edu.tw); Kuang-Yi Chang: [kychang@vghtpe.gov.tw](mailto:kychang@vghtpe.gov.tw);

Yih-Giun Cherng: [stainless@s.tmu.edu.tw](mailto:stainless@s.tmu.edu.tw); Shih-Pin Lin: [splin3@vghtpe.gov.tw](mailto:splin3@vghtpe.gov.tw);

Mei-Yung Tsou: [mytsou8095@gmail.com](mailto:mytsou8095@gmail.com)

Supplementary Table 1: Univariate analysis for recurrence-free survival (derivation cohort, n=1,008)

|  | **HR (95% CI)** | ***p*** |
| --- | --- | --- |
| **Age, year** | 1.013 (1.003 – 1.022) | 0.0071 |
| **Sex, male** | 1.207 (0.986 – 1.478) | 0.0689 |
| **Body mass index, kg·m^-2^** | 0.976 (0.945 – 1.007) | 0.1250 |
| **Cigarette smoking** | 1.118 (0.894 – 1.399) | 0.3284 |
| **ASA class ≥ 3** | 1.393 (1.118 – 1.736) | 0.0031 |
| **ECOG grade ≥ 1** | 1.458 (1.186 – 1.792) | 0.0003 |
| **Comorbidities** |  |  |
| Chronic obstructive pulmonary disease | 1.341 (0.985 – 1.825) | 0.0627 |
| Diabetes | 1.389 (1.085 – 1.777) | 0.0092 |
| Coronary artery disease | 0.879 (0.618 – 1.250) | 0.4724 |
| Heart failure | 1.496 (0.972 – 2.301) | 0.0669 |
| Stroke | 1.121 (0.657 – 1.912) | 0.6755 |
| Chronic kidney disease | 1.027 (0.707 – 1.491) | 0.8903 |
| **Preoperative pulmonary function** |  |  |
| FVC, % predicted | 0.493 (0.254 – 0.955) | 0.0361 |
| FEV1, % predicted | 0.410 (0.217 – 0.775) | 0.0061 |
| **Preoperative carcinoembryonic antigen, μg·L^-1^**† | 2.399 (1.899 – 3.030) | <.0001 |
| **Preoperative hemoglobin concentration, g·dL^-1^** | 0.893 (0.837 – 0.952) | 0.0005 |
| **Surgical and anesthetic variables** |  |  |
| Bilobectomy or pneumonectomy vs. sublobar resection or lobectomy | 1.409 (1.123 – 1.768) | 0.0030 |
| Thoracoscopic surgery | 0.784 (0.639 – 0.964) | 0.0207 |
| Radical lymph node dissection | 0.820 (0.595 – 1.131) | 0.2272 |
| Intraoperative blood loss, mL‡ | 1.173 (1.090 – 1.261) | <.0001 |
| Blood transfusion | 1.648 (1.285 – 2.115) | <.0001 |
| Epidural analgesia | 0.923 (0.703 – 1.213) | 0.5666 |
| Anesthesia time, min | 1.695 (1.260 – 2.279) | 0.0005 |
| **Inflammation biomarkers** |  |  |
| Prognostic nutritional index‡ | 0.127 (0.066 – 0.247) | <.0001 |
| Preoperative NLR‡ | 1.510 (1.326 – 1.720) | <.0001 |
| Postoperative NLR‡ | 1.089 (0.974 – 1.217) | 0.1334 |
| Absolute change of NLR‡ | 1.015 (0.924 – 1.116) | 0.7519 |
| Relative change of NLR, %‡ | 0.867 (0.799 – 0.940) | 0.0006 |
| Preoperative PLR‡ | 1.412 (1.198 – 1.665) | <.0001 |
| Postoperative PLR‡ | 1.121 (1.002 – 1.253) | 0.0455 |
| Absolute change of PLR‡ | 0.988 (0.927 – 1.054) | 0.7217 |
| Relative change of PLR, %‡ | 0.946 (0.891 – 1.006) | 0.0765 |
| Absolute change of lymphocyte count, 10^3^·μL^−1^ | 1.000 (1.000 – 1.000) | 0.1102 |
| **Pathologic features** |  |  |
| Cancer stage |  | <.0001 |
| II vs. I | 3.748 (2.884 – 4.872) | <.0001 |
| III vs. I | 5.754 (4.543 – 7.287) | <.0001 |
| Subtype |  | 0.0967 |
| SCC vs. adenocarcinoma | 1.339 (1.000 – 1.794) | 0.0503 |
| Others vs. adenocarcinoma | 1.259 (0.843 – 1.880) | 0.2601 |
| Tumor differentiation |  | <.0001 |
| Moderate vs. good | 6.411 (2.384 – 17.239) | 0.0002 |
| Poor vs. good | 16.352 (6.076 – 44.006) | <.0001 |
| Microscopic necrosis | 2.182 (1.766 – 2.696) | <.0001 |
| Lymphocytic infiltration | 1.282 (0.968 – 1.696) | 0.0829 |
| Lymphovascular invasion | 4.180 (3.407 – 5.130) | <.0001 |
| Perineural infiltration | 2.647 (1.776 – 3.946) | <.0001 |
| **Preoperative chemotherapy ± radiotherapy** | 2.868 (2.032 – 4.048) | <.0001 |
| **Postoperative chemotherapy** | 3.374 (2.691 – 4.231) | <.0001 |
| **Postoperative radiotherapy** | 3.474 (2.560 – 4.716) | <.0001 |
| **Year of operation (2015-11 vs. 2005-10)** | 0.946 (0.768 – 1.166) | 0.6027 |

HR: hazard ratio; CI: confidence interval. ASA: American Society of Anesthesiologists; ECOG: Eastern Cooperative Oncology Group; FEV1: forced expiratory volume in one second; FVC: forced vital capacity; NLR: neutrophil-to-lymphocyte ratio; PLR: platelet-to-lymphocyte ratio; SCC: squamous cell carcinoma. †On base-10 logarithmic scale; ‡On base-2 logarithmic scale

Supplementary Table 2: Univariate analysis for overall survival (derivation cohort, n=1,008)

|  | **HR (95% CI)** | ***p*** |
| --- | --- | --- |
| **Age, year** | 1.037 (1.021 – 1.053) | <.0001 |
| **Sex, male** | 2.078 (1.489 – 2.899) | <.0001 |
| **Body mass index, kg·m^-2^** | 1.010 (0.962 – 1.061) | 0.6895 |
| **Cigarette smoking** | 1.859 (1.346 – 2.567) | 0.0002 |
| **ASA class ≥ 3** | 2.128 (1.546 – 2.928) | <.0001 |
| **ECOG grade ≥ 1** | 2.905 (2.110 – 3.998) | <.0001 |
| **Comorbidities** |  |  |
| Chronic obstructive pulmonary disease | 2.123 (1.417 – 3.180) | 0.0003 |
| Diabetes | 1.512 (1.042 – 2.194) | 0.0296 |
| Coronary artery disease | 0.845 (0.488 – 1.463) | 0.5473 |
| Heart failure | 1.949 (1.081 – 3.514) | 0.0264 |
| Stroke | 2.522 (1.364 – 4.663) | 0.0032 |
| Chronic kidney disease | 1.545 (0.933 – 2.557) | 0.0909 |
| **Preoperative pulmonary function** |  |  |
| FVC, % predicted | 0.603 (0.216 – 1.685) | 0.3347 |
| FEV1, % predicted | 0.399 (0.147 – 1.087) | 0.0725 |
| **Preoperative carcinoembryonic antigen, μg·L^-1^**† | 3.121 (2.199 – 4.429) | <.0001 |
| **Preoperative hemoglobin concentration, g·dL^-1^** | 0.914 (0.825 – 1.011) | 0.0809 |
| **Surgical and anesthetic variables** |  |  |
| Bilobectomy or pneumonectomy vs. sublobar resection or lobectomy | 1.591 (1.131 – 2.236) | 0.0076 |
| Thoracoscopic surgery | 0.667 (0.480 – 0.928) | 0.0162 |
| Radical lymph node dissection | 0.794 (0.480 – 1.316) | 0.3714 |
| Intraoperative blood loss, mL‡ | 1.349 (1.203 – 1.514) | <.0001 |
| Blood transfusion | 2.362 (1.666 – 3.348) | <.0001 |
| Epidural analgesia | 0.663 (0.447 – 0.985) | 0.0418 |
| Anesthesia time, min | 3.166 (1.990 – 5.036) | <.0001 |
| **Inflammation biomarkers** |  |  |
| Prognostic nutritional index‡ | 0.059 (0.021 – 0.169) | <.0001 |
| Preoperative NLR‡ | 1.821 (1.493 – 2.220) | <.0001 |
| Postoperative NLR‡ | 1.195 (1.009 – 1.414) | 0.0389 |
| Absolute change of NLR‡ | 1.067 (0.920 – 1.237) | 0.3900 |
| Relative change of NLR, %‡ | 0.836 (0.738 – 0.946) | 0.0047 |
| Preoperative PLR‡ | 1.711 (1.330 – 2.200) | <.0001 |
| Postoperative PLR‡ | 1.215 (1.026 – 1.438) | 0.0240 |
| Absolute change of PLR‡ | 1.000 (0.902 – 1.108) | 0.9972 |
| Relative change of PLR, %‡ | 0.939 (0.853 – 1.033) | 0.1979 |
| Absolute change of lymphocyte count, 10^3^·μL^−1^ | 1.000 (1.000 – 1.000) | 0.9654 |
| **Pathologic features** |  |  |
| Cancer stage |  | <.0001 |
| II vs. I | 3.890 (2.581 – 5.864) | <.0001 |
| III vs. I | 5.088 (3.552 – 7.288) | <.0001 |
| Subtype |  | <.0001 |
| SCC vs. adenocarcinoma | 2.412 (1.641 – 3.544) | <.0001 |
| Others vs. adenocarcinoma | 1.494 (0.823 – 2.712) | 0.1870 |
| Tumor differentiation |  | <.0001 |
| Moderate vs. good | 4.656 (1.145 – 18.926) | 0.0316 |
| Poor vs. good | 9.815 (2.407 – 40.024) | 0.0014 |
| Microscopic necrosis | 2.759 (2.010 – 3.788) | <.0001 |
| Lymphocytic infiltration | 1.254 (0.831 – 1.893) | 0.2804 |
| Lymphovascular invasion | 3.832 (2.786 – 5.270) | <.0001 |
| Perineural infiltration | 1.401 (0.656 – 2.992) | 0.3833 |
| **Preoperative chemotherapy ± radiotherapy** | 2.291 (1.365 – 3.844) | 0.0017 |
| **Postoperative chemotherapy** | 1.936 (1.394 – 2.689) | <.0001 |
| **Postoperative radiotherapy** | 3.750 (2.457 – 5.723) | <.0001 |
| **Year of operation (2015-11 vs. 2005-10)** | 0.755 (0.525 – 1.085) | 0.1291 |

HR: hazard ratio; CI: confidence interval. ASA: American Society of Anesthesiologists; ECOG: Eastern Cooperative Oncology Group; FEV1: forced expiratory volume in one second; FVC: forced vital capacity; NLR: neutrophil-to-lymphocyte ratio; PLR: platelet-to-lymphocyte ratio; SCC: squamous cell carcinoma. †On base-10 logarithmic scale; ‡On base-2 logarithmic scale

**Supplementary Table 3: C-statistics of preoperative neutrophil-to-lymphocyte ratio for predicting recurrence and mortality in NSCLC with EGFR mutation (validation cohort, n=165)**

|  | **Continuous NLR** | |  | **Dichotomous NLR**† | |
| --- | --- | --- | --- | --- | --- |
|  | **C-statistic (95% CI)** | ***p*** |  | **C-statistic (95% CI)** | ***p*** |
| **1-Year Recurrence** | 0.509 (0.406 – 0.612) | 0.8515 |  | 0.565 (0.468 – 0.662) | 0.1876 |
| **3-Year Recurrence** | 0.561 (0.473 – 0.649) | 0.1792 |  | 0.580 (0.493 – 0.667) | 0.0756 |
| **5-Year Recurrence** | 0.551 (0.463 – 0.640) | 0.2607 |  | 0.571 (0.483 – 0.659) | 0.1221 |
| **1-Year Mortality** | 0.537 (0.460 – 0.613) | 0.8998 |  | 0.680 (0.312 – 1.000) | 0.5357 |
| **3-Year Mortality** | 0.658 (0.376 – 0.939) | 0.2310 |  | 0.625 (0.372 – 0.878) | 0.3418 |
| **5-Year Mortality** | 0.605 (0.433 – 0.777) | 0.2084 |  | 0.598 (0.434 – 0.762) | 0.2406 |

CI: confidence interval; NLR: neutrophil-to-lymphocyte ratio. † The cut-off value is 2.3 for both recurrence and mortality
